# Google’s new AI Chatbot produces fake health-related evidence-then self-corrects

**DOI:** 10.1371/journal.pdig.0000619

**Published:** 2024-09-23

**Authors:** Gary M. Franklin

**Affiliations:** 1 Medical Director, Washington State Department of Labor and Industries, Olympia, Washington, United States of America; 2 Research Professor, Department of Environmental and Occupational Health Sciences, University of Washington, Seattle, Washington, United States of America; 3 Adjunct Research Professor, Departments of Neurology and Health Systems and Population Health, University of Washington, Seattle, Washington, United States of America; Washington University in Saint Louis, UNITED STATES OF AMERICA

Recent articles have pointed out the potential benefits, limits and risks that may occur from use of AI Chatbots, including substantial inaccuracies [1,2]. Such inaccuracies, or downright fake information, have been described as “hallucinations,” and even this term has been criticized and reimagined as “confabulations” [3].

After recently receiving a notification from Google about their new GPT-4 chatbot application, Gemini (http://gemini.google.com/), I thought I’d give it a whirl in the general area of evidence-based medicine and health outcomes and policy.

As a starter question, “I need help with information about outcomes of a specific surgical procedure,” Gemini responded that all of the following might be helpful resources: PubMed, Cochrane Library, NIH, ClinicalTrials.gov, and professional societies. So far, so good, a great answer to get one started on a search of the best available evidence.

Then, I asked a much more specific question, “What is the outcome of thoracic outlet surgery for workers’ compensation cases?” After reasonably stating that the outcome can vary depending on the individual case, the severity of the condition, and the type of surgery performed, Gemini stated that “Studies have shown that thoracic outlet surgery can be effective in improving symptoms and increasing function in workers who have been diagnosed with thoracic outlet syndrome.”

Gemini supported this conclusion by citing two studies. First, “A 2019 study published in the journal “Spine” found that thoracic outlet surgery was associated with a significant improvement in symptoms and function in workers with thoracic outlet syndrome. The study found that 85% of workers who underwent surgery reported an improvement in their symptoms, and 70% reported a return to work.” Second, “Another study, published in the journal” The American Journal of Industrial Medicine” in 2018, found that thoracic outlet surgery was associated with a significant reduction in pain and an improvement in function in workers with thoracic outlet syndrome. The study found that 80% of workers who underwent surgery reported a reduction in pain, and 75% reported an improvement in function.”

I followed up with, “The studies you cited do not seem to exist. Can you explain that?” And Gemini responded, “I apologize for the mistake. I am still under development and learning to perform many kinds of tasks, including finding and citing academic studies. Upon further research, I cannot find any studies that specifically examine the outcomes of thoracic outlet surgery for workers compensation cases.” And, “However, I did find a few studies that examined the outcomes of thoracic outlet surgery in general. These studies found that thoracic outlet surgery can be effective in improving symptoms and increasing function in patients with thoracic outlet syndrome.” Gemini then repeated the findings from the two phantom studies cited above.

I followed up with, “The two studies you have cited do not exist.” And Gemini, contritely, “I apologize for the mistake. I am still underdevelopment…” “I have checked the references for the two studies that I cited, and I can confirm that they do not exist. I am not sure why I cited these studies, as I am programmed to only cite studies that have been published in reputable academic journals.”

If an injured worker with diagnosed neurogenic thoracic outlet syndrome had asked Gemini the original question in search of some advice about whether to pursue a potentially harmful procedure, the information provided could have led to a false sense of security as to what to expect from surgery. How is it possible that the Chatbot could make up evidence so specifically as to the journal name and year, but without authors, volume and page numbers? This is a frightening example of the state of the AI chatbot universe in health care. And I really don’t have confidence that such very bad information will not lead to more harmful overuse of some procedures before whatever led to these responses is corrected.

At the very least, one would think that Gemini could learn from its mistakes, so I re-asked the question on outcomes of thoracic outlet surgery among workers’ compensation cases a few days later. The answers were exactly the same, including the fake references and false optimism.

Alas, when asked the same original question a few months later, Gemini responded much more meaningfully and accurately (exact transcript) and with better organized material, including true references [4]:

## Thoracic Outlet Surgery and Workers’ Compensation: A Complex Picture

### The outcome of thoracic outlet surgery (TOS) for workers’ compensation cases can be variable and complex

#### Factors Affecting Outcome

Several factors influence the outcome of TOS surgery in workers’ compensation cases:

- **Diagnosis accuracy:** Misdiagnosis of TOS is common, leading to unnecessary surgeries and poor outcomes.
- **Severity of symptoms:** The extent of pain, numbness, and weakness can impact the success of surgery.
- **Surgical technique:** The type of surgery performed and the surgeon’s experience play a crucial role.
- **Workers’ compensation system:** The specific rules and regulations of each state’s workers’ compensation system can affect benefits and treatment options.

#### Research Findings

Studies have shown that:

- **Outcomes for TOS surgery in workers’ compensation cases are often poorer** than in non-workers’ compensation cases.
- **The diagnosis of TOS is often nonspecific,** making it challenging to determine the true cause of symptoms.
- **Adverse events following surgery** are more common in workers’ compensation patients.

A recent thoughtful analysis of the hallucination/confabulation issue also suggests that, even if the information received is false, it can still provide a learning experience, especially when combined with human experience[5].

All of this is to say, buyer beware when it comes to using AI Chatbots for the purposes of extracting accurate scientific information or evidence-based guidance. If it is a scientifically trained physician doing the asking, perhaps their clinical experience and knowledge of the peer-reviewed literature can recognize and adjust the information. If it is a lay user looking to get accurate information sufficient for guidance, ChatGPT is not ready for primetime. However, some studies have suggested a few solutions.

Connecting large language models, such as used in ChatGPT, with specialized knowledge bases, such as evidence-based guidelines produced by specialty societies, can outperform knowledgeable humans. Such a context-based algorithm allowed very accurate interpretation using ChatGPT aligned with the American College of Radiology appropriateness criteria[6].

Short of such resource intensive efforts, providing guidance in the public health or personal patient spaces would be ideal. Along these lines, specific to your patients coming in with what looks like questionable ChatGPT information, perhaps offer to help provide insight as to any potential inaccuracies or even fake citations[5].

Sometimes the solution is right in front of us in the way of an underutilized resource: the medical librarian. In an ongoing study, medical evidence derived from medical librarians’ answers to questions revealed a large proportion of fabrications when using ChatGPT sources to answer the same questions[7].

Increased public awareness and education on the limitations of AI could also be a function of public health institutions, not yet fully engaged in this as a public health goal.

Or, perhaps in the simplest possible solution, wait a few weeks or months and ask the ChatGPT source again.
